# The Immune Pathogenesis of Acute-On-Chronic Liver Failure and the Danger Hypothesis

**DOI:** 10.3389/fimmu.2022.935160

**Published:** 2022-07-14

**Authors:** Rui Qiang, Xing-Zi Liu, Jun-Chi Xu

**Affiliations:** ^1^ The Affiliated Infectious Diseases Hospital, Suzhou Medical College of Soochow University, Suzhou, China; ^2^ Key Laboratory of Oral Diseases Research of Anhui Province, College and Hospital of Stomatology, Anhui Medical University, Hefei, China; ^3^ Key Laboratory of Infection and Immunity of Suzhou City, The Fifth People’s Hospital of Suzhou, Suzhou, China

**Keywords:** ACLF, DAMPs, danger hypothesis, inflammatory cytokine storm, biological target molecules

## Abstract

Acute-on-chronic liver failure (ACLF) is a group of clinical syndromes related to severe acute liver function impairment and multiple-organ failure caused by various acute triggering factors on the basis of chronic liver disease. Due to its severe condition, rapid progression, and high mortality, it has received increasing attention. Recent studies have shown that the pathogenesis of ACLF mainly includes direct injury and immune injury. In immune injury, cytotoxic T lymphocytes (CTLs), dendritic cells (DCs), and CD4^+^ T cells accumulate in the liver tissue, secrete a variety of proinflammatory cytokines and chemokines, and recruit more immune cells to the liver, resulting in immune damage to the liver tissue, massive hepatocyte necrosis, and liver failure, but the key molecules and signaling pathways remain unclear. The “danger hypothesis” holds that in addition to the need for antigens, damage-associated molecular patterns (DAMPs) also play a very important role in the occurrence of the immune response, and this hypothesis is related to the pathogenesis of ACLF. Here, the research status and development trend of ACLF, as well as the mechanism of action and research progress on various DAMPs in ACLF, are summarized to identify biomarkers that can predict the occurrence and development of diseases or the prognosis of patients at an early stage.

## Highlights

- Immune damage is an important factor in the occurrence of ACLF, but the immune mechanism is unclear.
- The DAMP cycle and immune damage are important factors leading to cytokine storms.
- According to the “danger hypothesis,” DAMP release, inflammatory cytokine storms, and the occurrence of ACLF are closely related.
- Intrahepatic infiltration and hyperfunction of immune cells caused by DAMPs are important factors leading to the occurrence of ACLF.
- DAMPs can be biological target molecules for the early diagnosis and treatment of ACLF.

As a group of complex clinical syndromes related to severe acute liver function impairment and multiple-organ failure caused by various acute triggering factors on the basis of chronic liver disease, ACLF is characterized by severe disease, rapid progression, and high mortality. Currently, there are four categories of liver failure worldwide, namely, acute liver failure (ALF) (1), subacute liver failure (SALF) (2), ACLF (3), and chronic liver failure (CLF) (4), among which ACLF is a common type of liver failure. Additionally, chronic viral hepatitis, drug-induced hepatitis, and alcoholic hepatitis are major diseases induced by ACLF.

Immune damage is an important mechanism for the occurrence of ACLF. Recent studies have shown that intrahepatic immune cell infiltration and cytokine storms are important factors, but the key molecules involved in immune hyperfunction in ACLF remain unclear. “The danger hypothesis” holds that in addition to the antigens that the immune system responds to when detected, the DAMP-mediated injury-hyperimmunity cycle coincides with the mechanism of ACLF; thus, DAMPs may be closely related to the occurrence of ACLF. This paper summarizes the research status and development trends related to ACLF, as well as the mechanism of action and research progress on various DAMPs in ACLF.

## 1 The definition, inducement, treatment, research status, and development trends of ACLF

### 1.1 The Controversial Definition of ACLF

Liver failure is a group of clinical syndromes in which severe liver damage caused by many factors results in severe dysfunction or decompensation of synthesis, detoxification, metabolism, and biotransformation, with jaundice, coagulation disorders, hepatorenal syndrome, hepatic encephalopathy (HE), and ascites as the main manifestations (5). ACLF, as the most common severe liver disease syndrome in clinical practice, has various causes, complex clinical manifestations, and high mortality.

Currently, the definitions of ACLF in different areas of research are controversial, which mainly results from differences in patients in Europe, America, Asia Pacific, and other regions, as well as medical histories, diagnostic criteria, and acute inducing factors. The European Association for the Study of the Liver-Chronic Liver Failure (EASL-CLIF) defines ACLF as a severe syndrome in patients with liver cirrhosis that has three typical characteristics: acute liver decompensation, organ failure, and short-term high mortality. This definition not only includes intrahepatic symptoms but also reflects the damage caused by ACLF to multiple organs or systems including the liver and other tissues (6). The North American Consortium for the Study of End-Stage Liver Disease (NACSELD) defines ACLF as involving two or more extrahepatic organ failures, excluding changes in liver function and the coagulation system (7). The definition of ACLF by the Asian Pacific Association for the Study of the Liver (APASL) was published in 2009 (8) and updated in 2014 (9) and 2019 (10), including patients with liver cirrhosis (compensatory period) or chronic liver disease who have or not been diagnosed in the past, acute liver injury complicated with jaundice (serum bilirubin ≥ 5 mg/dl (85 μmol/l), and coagulation disorders (international normalized ratio ≥ 1.5 or prothrombin activity < 40%), ascites and/or HE within 4 weeks; no history of decompensation and extrahepatic sediment, renal failure, and circulatory or respiratory failure are excluded from the definition, and the mortality rate within 28 days is usually high.

### 1.2 Inducement and Treatment Status of ACLF

According to recent clinical studies, the main causes of death in ACLF patients include hepatitis virus reactivation, sepsis caused by bacterial infection, severe alcoholic liver disease, and drug toxicity and side effects.

Currently, the treatment of ACLF generally includes medical support treatment, artificial liver treatment, and liver transplantation treatment, but ACLF is still characterized by severe illness, rapid progression, and high mortality in the clinic (5). Treatment studies of liver failure have been continuously developed to further clarify the pathogenesis of ACLF, explore related factors of its prognosis, find early diagnosis and treatment targets, predict patient outcomes such that clinical intervention treatment can be carried out in a timely manner, improve prognosis, and reduce mortality.

### 1.3 Research Status and Development Trends of the Immune Pathogenesis of ACLF

At present, the pathogenesis of ACLF remains unclear, although recent studies have shown that the pathogenesis of ACLF mainly includes direct injury and immune injury. Pathogen-associated molecular patterns (PAMPs) released by pathogens themselves or DAMPs induced by various factors bind to receptors and stimulate the release of proinflammatory cytokines such as interleukin (IL)-1β, IL-6, and IL-8, leading to immune disorders and thus causing a “cytokine inflammatory storm,” sepsis, tissue hypoperfusion, and mitochondrial dysfunction, all of which can culminate in multiple-organ failure and ACLF (11–13).

From the perspective of immune cell function, cellular immunity mediated by CTLs is the main factor causing massive hepatocyte necrosis (14). Studies have shown that IFN-γ and TNF-α expression in the liver of ACLF patients is markedly upregulated, which is significantly related to accumulation of CD4^+^ and CD8^+^ T cells (15). Some studies have found that the expression level of programmed cell death protein 1 (PD-1) on CD8^+^ T cells decreases significantly in the early stage of ACLF, which diminishes the negative regulation of CTL immune activity and promotes disease aggravation (16). In addition, some studies have found that DCs migrate from the blood to the liver in ACLF patients, inhibiting IFN-γ secretion by CD8^+^ T cells and reducing liver damage (17). CD4^+^ T cells are also an important factor in the occurrence of ACLF. Compared with healthy people and chronic hepatitis B (CHB) patients, regulatory T cells (Tregs) in the peripheral blood of ACLF patients are significantly increased, which correlates positively with the viral nucleic acid load, suggesting that it may be related to disease severity (18). Other studies have observed immune imbalance between Th17 and Treg cells in ACLF patients. When ACLF occurs, there are asynchronous increases in Th17 cells and decreases in Treg cells, and prognosis is poor when the ratio of Treg/Th17 cells is low (19, 20). Some scholars have studied the number and function of peripheral blood mononuclear cells in the course of ACLF, with human leukocyte antigen–DR isotype (HLA-DR) showing a downward trend with disease aggravation; the proinflammatory factors IL-1β and TNF-α secreted by monocytes are also increased in the early stage and decreased in the late stage. The immune state of the body changes from a severe proinflammatory reaction at the beginning of the disease to an anti-inflammatory reaction stage, which enhances the risk of infection and further aggravates the disease (21). Moreover, some scholars have put forward the “triple attack theory,” which suggests that the occurrence of liver failure includes a triple attack of immune injury, ischemia, and hypoxia injury and endotoxemia. On this basis, it is proposed that immunotherapy for ACLF should be carried out in stages, that is, immunosuppressive therapy should be given at the initial stage and immune enhancement therapy should be given at the middle and late stages (22).

In addition to various immune cells, many cytokines are involved in the occurrence and development of ACLF. Compared with acute hepatitis B patients, CHB patients, and healthy controls, the serum IL-1Rα level in HBV-ACLF patients is significantly increased, although the ratio of IL-1Rα/IL-1β is significantly decreased. Further follow-up has shown that the level of IL-1Rα and the IL-1Rα/IL-1β ratio are closely related to survival time, and many proinflammatory cytokines, such as IL-6, IL-8, and IL-23, are significantly related to the occurrence and prognosis of ACLF (14, 20, 21).

In summary, a variety of immune cells and cytokines are involved in the pathogenesis of ACLF. In the early stage, CTLs, DCs, and CD4^+^ T cells accumulate in the liver tissue, secrete a variety of proinflammatory cytokines and chemokines, and recruit more immune cells to the liver, resulting in immune damage to the liver and leading to massive hepatocyte necrosis and liver failure. In general, the key factors leading to aggravation of immune injury can be identified by further understanding the immune mechanism of ACLF, which will provide an important theoretical basis for early treatment of liver failure and curb its further development.

## 2 Types and functions of inflammatory factors related to the danger hypothesis

**Figure 1:**
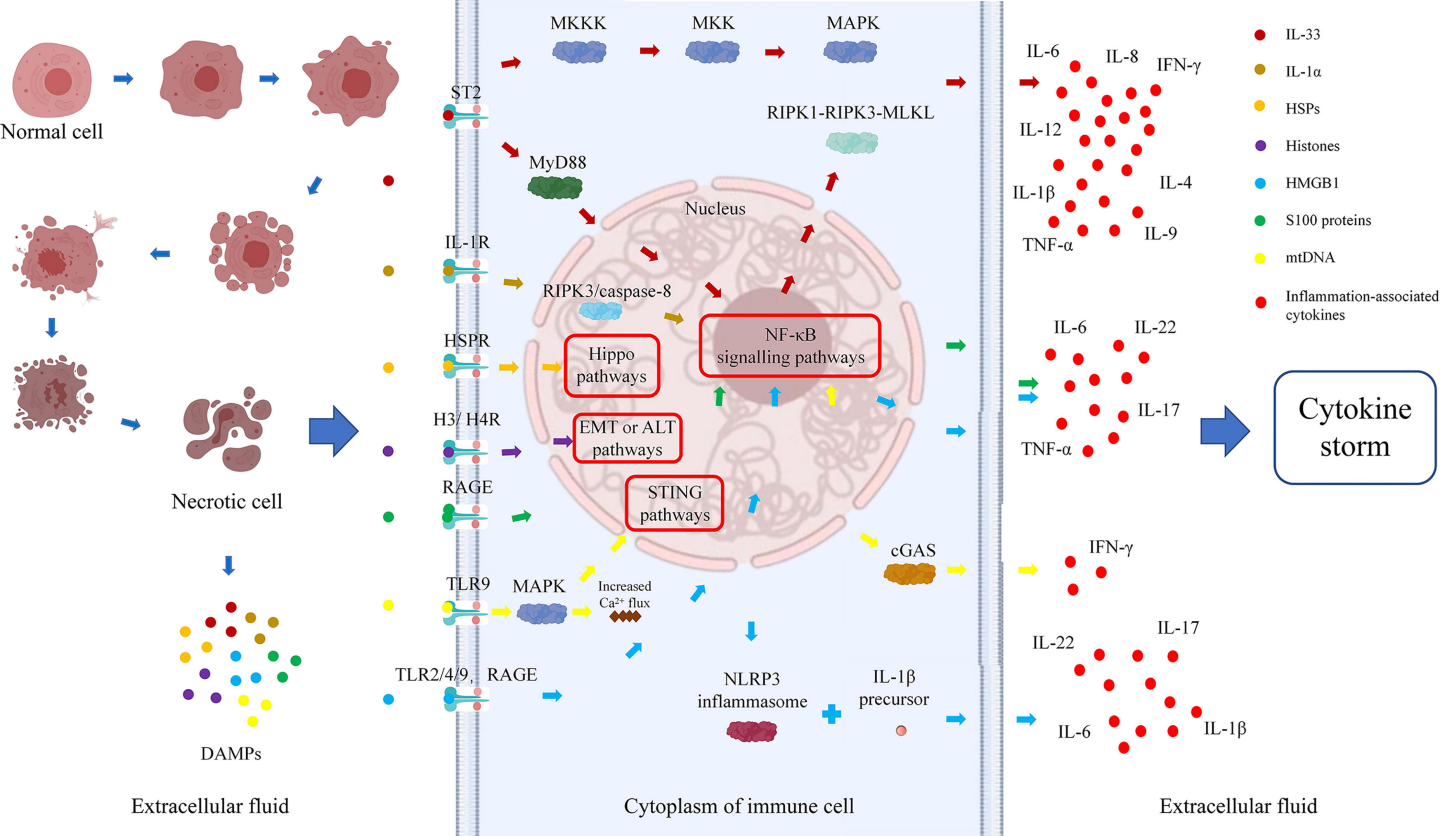
Danger signal cycle: relationships among cell necrosis, DAMPs, and cytokine storms.

Dr. Matzinger first put forward the “danger hypothesis” in 1994, which holds that the immune system will respond only when it detects danger; otherwise, it is immune tolerant (23). Subsequently, Dr. Matzinger put forward the “danger model” in 2002, which shows that the immune system is more concerned about the danger signals generated by cell damage or stress, than with unconditionally recognizing and removing foreign substances. Regardless of whether danger signals are exogenous pathogens released from necrotic cells or chemicals synthesized or modified by endogenous cells themselves, they can trigger immune responses through tissue damage or cell stress and are not emitted by healthy cells or cells in a normal physiological death state (24). Necrosis of cells can result in the generation of danger signals, which will promote secretion of cytokines by immune cells, further aggravate immune damage to cells, form a cycle of tissue damage, and lead to the generation of a cytokine storm and massive tissue necrosis (**Figure 1**).

There is no clear definition of the properties of danger signaling molecules, which can be divided into physical, chemical, and biological molecular forms according to different inducements. A large class of danger signaling molecules, DAMPs belong to the immune stimulation molecular model in aseptic inflammation, which is different from the microbial model and is usually related to injury (25). DAMPs are produced by necrotic and stressed cells or tissues and can bind to host pattern recognition receptors (PRRs) and other receptors, transmitting proinflammatory signals and promoting secretion of inflammatory factors and the inflammatory response. DAMPs are mainly derived from the nucleus, cytoplasm, organelles, or extracellular matrix and include uric acid, mitochondrial DNA (mtDNA), ATP, heat shock proteins (HSPs), amyloid β, S100 protein, high-mobility group box chromosomal protein 1 (HMGB1), ECM protein, IL-1α, IL-33, genomic DNA, cyclophilic protein A, fibrous actin (F-actin), and calreticulin (26, 27). DAMPs are associated with LF occurrence and development. All studies of IL-33, IL-1α, S100 protein, and mitochondrial DNA and 75% of those of histones have shown that DAMP expression is elevated during ACLF and correlates positively with disease severity. Studies of HSPs and 25% of studies of histones found that expression is decreased, which might prevent the occurrence of ACLF (**Table 1**). Therefore, analysis of the relationship between DAMPs and ACLF may be of great significance to elucidate the pathogenesis of ACLF.

**Table 1:** DAMPs associated with ALF.

Intracellular location	DAMPs	NOT ACLF		ACLF	
		(ALF/ALI)			
		Upregulation	Upregulation	Upregulation	Upregulation
		(protective)	(damaging)	(protective)	(damaging)
**Nucleus**	IL-33	Animal models:	Animal models:		Humans: (28–34)
		(35–38)	(35, 39–41)		
	IL-1α		Animal models: (44–48)		Humans: (42, 43)
					Animal model: (43)
	Histones		Humans: (49, 50)	Animal model: (53)	Humans: (51, 52)
			Animal models:		Animal model:
			(49, 50, 54)		(55)
	HMGB1		Humans: (56–59)		Humans: (56, 60)
			Animal models:		Animal models:
			(61–66)		(66–68)
**Cytosol**	HSPs	Humans: (69–71)	Animal models: (73–77)	Humans: (72)	
		Animal models:		Animal model:	
		(69, 70, 78–90)		(87)	
	ATP			Humans: (91, 92)	
				Animal models:	
				(91, 92)	
	S100 Protein		Humans: (93, 94)	Humans: (58, 95, 96)	
			Animal model:	Animal model:	
			(97)	(98)	
**Mitochondria**	Mitochondrial DNA		Human model:		Animal model:
			(99)		(100, 101)

## 3 Types and functions of DAMPs related to ACLF

### 3.1 Interleukin-1 Family Cytokines

In recent years, increasing attention has been given to the relationship between the function of IL-1 superfamily members and the occurrence of ACLF. IL-1 superfamily members are involved in many inflammatory diseases, such as obesity, cardiovascular diseases, cancer, viral or parasitic infections, autoinflammatory syndrome, and liver diseases (102). The role of the IL-1 superfamily in liver diseases can be protective or proinflammatory, and two members, namely, IL-33 and IL-1α, which have been studied the most, are considered bifunctional cytokines. In addition to their functions as classical cytokines, as part of the DAMP process, IL-33 and IL-1α are early warning signals of cell injury (102). The IL-33/ST2 pathway has a bidirectional regulatory role in acute hepatocyte injury and chronic liver fibrosis, and the IL-1α/IL-1R1 axis promotes massive necrosis of hepatocytes and infiltration of inflammatory cells into liver tissue, leading to organ failure. Such enhanced immune cell tissue infiltration and cytokine secretion abilities may play an important role in liver immune injury.

Knowledge of effects of the IL-33/ST2 axis and IL-1α/IL-1R1 axis on ACLF will be helpful to reveal the pathogenesis of ACLF to provide a new target for early diagnosis and treatment of ACLF.

#### 3.1.1 Interleukin-33

IL-33 is a member of the IL-1 superfamily. As a tissue-derived nuclear cytokine, it is mainly derived from endothelial cells, epithelial cells, and fibroblasts during inflammation. Its receptor ST2 is a member of the Toll-like receptor superfamily and is expressed on the surface of many immune cells, such as mast cells, type II intrinsic lymphocytes (ILC2s), Tregs, helper T cells, CD8^+^ T cells, natural killer cells, B cells, and macrophages (103).

Kotsiou et al. summarized previous studies and found that acute large-scale liver injury could result in release of IL-33 from cells, which might be an activator of tissue self-protection and repair (35) or an anti-inflammatory factor marker of M2 macrophages (36). However, IL-33 acts as a liver fibrosis factor to aggravate liver deterioration in chronic liver injury (36). Rickard et al. found that IL-33 released from mouse liver necrosis tissue into the microenvironment forms a coupling complex with myeloid differentiation factor 88 (MyD88), IL-1, and a subset of Toll-like receptors; this process might be regulated by the RIPK1–RIPK3–MLKL axis and drive inflammation (39). Arshad et al. reported that the expression of IL-33 in hepatocytes is partly controlled by perforin and tumor necrosis factor-related apoptosis-inducing ligand (TRAIL) under the regulation of natural killer T cells but that is not controlled by tumor necrosis factor α (TNF-α) or Fas ligand (FasL). IL-33-deficient mice exhibit more severe liver injury than WT mice, suggesting that IL-33 has a protective effect against acute liver injury (37). According to Volarevic et al., acute liver injury induced by concanavalin A can be reduced by activating the IL-33/ST2 axis. ST2-deficient mice developed more severe hepatitis and more liver inflammatory cell infiltration, suggesting the potential of the IL-33/ST2 axis as a therapeutic target (38).

Nevertheless, some studies have shown that the IL-33/ST2 axis has a role in promoting the development of acute liver injury. Kim et al. (40) and Seo et al. (41) found serum levels of HMGB1 and IL-33 to be significantly increased in ALF animal models induced by D-galactosamine (GalN) and lipopolysaccharide (LPS) and that blocking the pathway with inhibitors (such as necrostatin-1) could reduce liver injury. IL-33/ST2 promotes activation of the nuclear factor (NF)-κB, mitogen-activated protein kinase (MAPK), or extracellular signal-regulated kinase (ERK)/p38-MAPK pathways to produce proinflammatory cytokines (IL-6, IL-8) and T helper type 2 (Th2)-related cytokines (IL-4, IL-5, IL-9, IL-13) (104, 105).

At present, there are few studies on the IL-33/ST2 axis in ACLF. Roth et al. found through serological experiments that compared with healthy people, the concentrations of IL-33 and soluble ST2 (sST2) in ALF and ACLF patients are significantly increased, which is helpful for distinguishing acute and chronic liver failure or for monitoring the progression and severity of the disease (28). Subsequently, immunohistochemistry by Lei et al. showed a weak IL-33 expression and high sST2 expression in liver slices of HBV-ACLF patients, suggesting that sST2 can be used as a predictor of disease severity (29). Jiang et al. found no significant difference in serum IL-33 among HBV-ACLF, CHB, and HC groups, even though serum sST2 levels were higher in HBV-ACLF patients, correlated with the survival rate, and decreased after treatment. It was suggested that sST2 can be used as a marker for evaluating disease severity and early recognition (30). In the study of Du et al., the expression level of IL-33/ST2 was significantly increased in the serum and liver tissue of ACLF patients, and the level of serum IL-33 was found to be related to the severity of liver disease. *In vitro* experiments proved that IL-33 enhanced the LPS-stimulated inflammatory storm of monocytes through ERK1/2 activation but not p38 and JNK activation (31). Regarding the course of ACLF caused by CHB, Yuan et al. showed that compared with levels in the CHB group and the pre-ACLF group, serum IL-33 and sST2 were highest in the ACLF group, which could be used to evaluate progression and mortality in patients with multiple biological indicators (32). Gao et al. showed that serum IL-33 and sST2 were highly expressed in HBV-ACLF and that sST2 may be used as a prognostic marker (106).

In conclusion, abundant clinical evidence and experimental data show that the IL-33/ST2 pathway is related to the occurrence and development of various acute liver diseases, but the role of the IL-33/ST2 axis in ALF/ACLF is still controversial. As a DAMP, IL-33 stimulates the immune system to respond to virus invasion through its receptor ST2, drives immune cell infiltration into the liver, increases secretion of cytokines, and causes toxic damage to liver cells. However, some studies have found that the IL-33/ST2 axis can also promote Th2-type responses and hepatic stellate cell activity, promoting the progression of liver fibrosis due to chronic damage, which is a protective mechanism.

#### 3.1.2 Interleukin 1α

IL-1α, a member of the IL-1 superfamily, is released by RIPK3/caspase-8 apoptosis signal transduction in a caspase-1-dependent manner during cell injury or apoptosis, from macrophages undergoing TNF-induced necroptotic death or from apoptotic bodies. IL-1α can trigger an inflammatory response regardless of cell damage or apoptosis (107). The receptors, the IL-1 receptor (IL-1R) family including more than 10 structurally related members such as IL-1R1, IL-1R2, and IL-1R3, which transmit inflammatory signals downstream, induce the accumulation of immune cells and promote the secretion of inflammatory cytokines. IL-1Rs are alert receptors that play a central role in sensing maladaptive tissue changes from both outside and within the body (108). One study found that IL-1R-/- mice had a higher survival rate than wild-type mice during chronic infection, which was due to the attenuated inflammatory response in the former, allowing them to recover from cachexia (109). As a common proinflammatory factor, IL-1α has been intensely studied in liver injury, mainly with regard to ALF and less so ACLF. In addition, many studies on the IL-1α/IL-1R axis in liver disease have high patient numbers.

Romics et al. used LPS to induce liver injury in mice and found that various serum proinflammatory cytokines (including IL-1α) were significantly increased and that mRNA expression levels of various proinflammatory cytokines in the liver also increased (44). Gehrke et al. showed that inhibiting IL-1R1 by blocking it with IL-1ra can alleviate the severity of ALF but that administration of IL-1α aggravates the degree of liver injury (45). Xiao et al. found that IL-1α can obviously induce and maximize a reduction in the liver inflammatory state (46). *Lactobacillus reuteri* DSM 17938 is a potential probiotic for preventing or treating liver failure. In one study, the serum IL-1α level, the inflammatory state, and acute liver injury decreased significantly in a rat model treated with *Lactobacillus reuteri* DSM 17938 (47). Sultan et al. reported that IL-1α plays a central role in the pathogenesis of fulminant liver failure in mice; the symptoms of ALF in IL-1α^-/-^mice were obviously alleviated, and secretion of inflammatory factors was obviously reduced (48).

Systemic inflammation can easily lead to ACLF, which may be related to activation of inflammatory corpuscles. The research of Monteiro and studies of other animal models have confirmed that development of ACLF in compensated cirrhosis is related to increases in IL-1α and IL-1β (42). Recently, a genetic study found two kinds of gene cluster polymorphisms belonging to the IL-1 superfamily, in which expression of IL-1α-related genes significantly reduced the occurrence of ACLF (43).

In conclusion, it may be a possible approach to treat ALF/ACLF by blocking the IL-1α/IL-1R axis.

### 3.2 Heat Shock Proteins

HSPs, which are produced by heat shock, ischemia, hypoxia, and other stress factors, participate in the correct folding, modification, and maturation of proteins; assist in the degradation of aging proteins; are expressed during cell stress; and play an important role in regulating antigen presentation, inflammatory signal transduction, and apoptosis (110). HSPs are classified according to their molecular weights as HSP25, HSP27, HSP60, HSP70, HSP90, HSP110, and glucose-regulated protein (GRP), among others. HSPs play an important role in the proliferation, invasion, metastasis, and apoptosis evasion of cancer cells and promote the development of diseases. As a new cancer diagnostic marker and therapeutic target, HSPs play an important role in evaluating the molecular mechanism of cancer development and metastasis (111). Overall, HSPs are very important for cell survival under unfavorable environmental conditions.

HSP27 overexpression is closely related to the tumorigenesis, metastasis, and invasiveness of cancer. For example, the elevated expression of HSP27 increases Salvador–Warts–Hippo pathway (Hippo pathway) nuclear localization, activates related oncogenic and metastatic pathways, including the TGF-B/SMAD, WNT/B-Catenin, and ILK signaling pathways, and leads to increased tumor cell expansion in local tissues (112). HSP70 induces cell proliferation, inhibits apoptosis and oncogene-induced senescence, and is a poor prognostic marker for various cancers (112). HSP70 promotes tumor metastasis and infiltration by upregulating the expression of molecules such as N-cadherin, MMP2, SNAIL, and vimentin (113). To date, there are few studies on the mechanisms of HSPs in ACLF, and those that have been conducted are mainly related to ALI and ALF.

HSPs have been considered to be protective factors in the occurrence of acute liver injury or liver failure. Oda et al. found in 2002 that geranylgeranylacetone (GGA) could prevent ALF after large-scale hepatectomy by inducing and enhancing HSP70 expression in residual liver (78). Subsequently, Kanemura et al. (79) and Kawashima et al. (80) reported that GGA initiated strong cell protection by inhibiting the CXC chemokine GRO1 and inducing HSP27 and HSP70. Sepsis causes acute inflammatory reactions in the liver, which can lead to organ failure and death. According to Peppler et al., exercise has anti-inflammatory and hepatoprotective effects; regular exercise increases liver protein levels, including HSP70, prevents the inflammatory cascade reaction induced by LPS, and weakens the severe inflammatory reaction of the liver caused by sepsis (81). Sumioka et al. showed that HSP25 and HSP70i have protective effects on acute liver injury induced by acetaminophen (APAP) in mice, and their levels might be key in determining the fate of APAP-injured mice (82). ALF induced by APAP damages the mitochondria and activates HSP70 expression, whereas diphenyl diselenide prevents ALF induced by APAP and plays a key role in regulating the cell protection response (83). Excessive production of HSP70 in the liver protects hepatocytes under various pathological conditions. It has been found that prostaglandin E1, a non-toxic HSP inducer, prevents ALF after large-scale hepatectomy by enhancing the production of HSP70 in residual liver (84). Bicyclic alcohol is a new type of hepatoprotective agent that induces the production of heat shock transcription factor 1 (HSF1) and promotes synthesis of HSP70 and the stress response, inducing sex-specific liver protection to prevent liver injury or failure (85). Dai et al. found that bicyclol induces the overexpression of HSP27 in the liver, which significantly enhances the protection of an animal model of acute liver injury induced by D-galactosamine/lipopolysaccharide (86). In the study of Vidyasagar et al., serum HSP25 and HSP27 had antioxidant effects in ALF and CLF patients and reduced the damage caused by reactive oxygen species (ROS) and prevented the occurrence of HE (69). El-Baz et al. showed that *D. salina* hydrochloride increased HSP25 and improved brain histopathological changes in HE patients, benefiting ALF prognosis (70).

Most scholars believe that HSPs are important protective factors in the repair of liver tissue damage and that increases in their levels are helpful for the prognosis of patients and predicting efficacy, but the mechanism of some remains controversial. Ye et al. found that HSP27 accelerated the early acute liver injury induced by ischemia–reperfusion injury in rats by reducing the number of Treg cells, reducing levels of the stress-protective factors superoxide dismutase (SOD) and glutathione, increasing the level of proinflammatory factors, and aggravating inflammation (73). Wright et al. conducted ACLF-related animal experiments and showed increased HSP25 in the corpus callosum of the bile duct ligation (BDL) rat model, suggesting a cell stress state (such as inflammation and apoptosis) (87).

Other members of the HSP family also play important roles in the development of acute liver injury or liver failure. Some studies have found that GRP78 combined with other preparations can significantly improve the incidence of ALF or prevent aggravation of acute liver injury. GRP78, a member of the HSP70 family, is a molecular chaperone for endoplasmic reticulum stress and stress-induced autophagy. Win et al. showed that Japanese miso extract prepared from rice yeast can enhance the expression of GRP78, inhibiting hepatitis A virus (HAV) replication and reducing ALF (88). Nwe et al. reported that free fatty acids or high concentrations of glucose enhance HAV replication and reduce GRP78 expression but that thapsigargin has the opposite effects, reducing the occurrence of ALF (71). The protective effect of kaempferol in the ALF mouse model occurs through inhibition of hepatocyte apoptosis by increasing the expression of GRP78 (89). Ren et al. found that compared with healthy people and patients with CHB, the expression of GRP78 and GRP94 in ACLF patients decreases gradually, indicating that HSP-mediated stress protection is decreased in ACLF patients and correlates negatively with the degree of liver injury (72).

However, some studies have reported that GRP78 and GRP94 are important factors for acute liver injury or liver failure. Baudi et al. found that acute liver injury induced by IFN-α-mediated virus infection could be alleviated by inhibiting GRP78 through a mechanism that involves IFN-α induction of ER stress-related cell death by reducing the unfolded protein response (UPR), with GRP78 promoting high UPR expression and reducing IFN-α-mediated liver injury in mice (74). Zhang et al. found that peroxisome proliferator-activated receptor α (PPARα) can improve liver injury caused by ALF; the mechanism is mainly to reduce hepatocyte apoptosis by regulating endoplasmic reticulum stress and reducing the expression of GRP78, GRP94, and other proteins (75). According to Blas-Valdivia et al., hypothyroidism reduces cell damage caused by endoplasmic reticulum stress and redox environment changes in an ALF rat model, which might be due to inhibition or decreased protein activation pathways, such as GRP78 (76).

Finally, there are two common HSPs, HSP40 and HSP60, that act as injury factors during acute liver injury or liver failure and play an important role in regulating the acute inflammatory response of the liver. A long-term treatment with isoniazid leads to severe liver injury and ALF. Verma et al. reported the following: the mechanism is that isoniazid prevents Nrf2 translocation and induces oxidative stress and apoptosis by inhibiting ERK1 phosphorylation, thus increasing levels of the stress proteins HSP40 and HSP60 (114). Hu et al. found that chlorogenic acid (CGA) reduces the infiltration of immune cells in the liver, prevents increases in HMGB1 and HSP60, and regulates the Nrf2-mediated HSP60 pathway to alleviate acute liver injury induced by acetaminophen in mice (77).

Studies of the HSP family in ALF/ACLF are relatively scarce, and the role of HSPs in the process of liver tissue injury remains unclear. Due to the lack of basic experiments, more research on HSPs is needed to clarify the mechanism.

### 3.3 Histones

Histones are an important structural component of eukaryotic chromatin that help regulate gene transcription. Histones are considered key mediators of systemic inflammatory diseases; induce endothelial injury and platelet aggregation and activate coagulation and cytokine production; and may cause sepsis, severe trauma, vasculitis, and acute liver, kidney, brain, and lung injury (115). Silvestre-Roig et al. found that the extracellular histone H4-mediated membrane lysis of smooth muscle cells (SMCs) triggers arterial tissue damage in atherosclerotic mice and attracts neutrophils to exacerbate the inflammatory response and that neutralizing histone H4 can prevent SMC death and stabilize atherosclerotic lesions (116). Ray-Gallet et al. summarized the potential oncogenic roles of histones H3 and H4 and their chaperones in several cancers, indicating that they stimulate the epithelial–mesenchymal transition (EMT) in various ways or the alternative lengthening of telomeres (ALT) pathway to promote tumor progression, illustrating their potential clinical application as biomarkers (117). Despite increasing attention on histone H3, only a few studies have explored the importance of H4 and its chaperones or ways to inhibit their action as a new therapeutic strategy (118).

In recent years, increasing attention has been given to the role of histones as DAMP molecules in acute liver injury, ALF, and ACLF to find potential new biomarkers and therapeutic targets. Extracellular histones, especially H4, have been recognized as important mediators of cell injury under various inflammatory conditions. High expression of extracellular histones is closely related to the development of inflammation in acute liver injury and acute liver failure.

In the ALF mouse model of Ferriero et al., pyruvate dehydrogenase complex (PDHC) and lactate dehydrogenase (LDH) are transferred to the nucleus, which leads to an increase in acetyl coenzyme A and lactic acid concentrations in the nucleus and promotes acetylation of histone H3 and expression of injury-related genes. However, liver injury in ALF mice can be reduced and the survival rate improved by the enzyme inhibitors gamboge and galloflavin (54). Wen et al. (49) and Yang et al. (50) both showed that extracellular histones increase to different degrees in ALF patients and ALF mice, demonstrating that extracellular histones are the main mediator inducing systemic inflammation, cell damage, and multiple-organ failure. Clinical studies by Li et al. highlighted that compared to levels in chronic hepatitis B (CHB), liver cirrhosis, and healthy control groups, plasma histone H4 levels in HBV-ACLF patients are significantly increased, aggravating cell injury and systemic inflammation, and are significantly related to disease severity, systemic inflammation, and outcomes (51). Ding et al. found that the Qing Chang Li Gan formula (QCLGF) is a good traditional Chinese medicine for treating ACLF, which may be due to its ability to interfere with extracellular histone-mediated cell damage and systemic inflammation. In one study, extracellular histones and proinflammatory cytokines in ACLF patients (conventional drugs + QCLGF) were lower than those in the conventional drug treatment group. *In vivo* experiments have revealed that QCLGF significantly improves the survival rate of concanavalin A-induced liver fibrosis mice, improves hepatotoxicity, and reduces extracellular histone levels and proinflammatory reactions (55).

Histone methylation, phosphorylation, and acetylation are markers of gene transcriptional status in several diseases, and different posttranslational modification patterns are related to some inflammatory diseases (119). Jin et al. found that the whole-genome histone H3 lysine 9 acetylation analysis of CD4^+^ T cells indicated endoplasmic reticulum stress defects in ACLF patients, which provided a useful clue for further study of the pathogenesis of ACLF (52). Zhang et al.’s research showed that trichostatin A (TSA) reduces the activity of histone deacetylase inhibitors (HDACs) in liver tissue, promotes histone acetylation, inhibits release of various proinflammatory cytokines (TNF-α, IFN-γ, IL-10, and IL-18), and improves the survival rate of ACLF model rats (53).

Although studies of extracellular H3 and H4 in ALF and ACLF are rare and the mechanism is still unclear, some experiments have shown that extracellular histones play an irreplaceable role in liver injury. The importance of histones as proinflammatory proteins in ALF and ACLF should be further explored.

### 3.4 High-Mobility Group Box Chromosomal Protein 1

HMGB1 is a non-histone chromatin-related protein that is widely distributed in eukaryotic cells. A DAMP, HMGB1 is actively secreted by immunocompetent cells or passively released from apoptotic necrotic cells, which activates the immune response and promotes inflammation and cancer development (120). HMGB1 transmits danger signals by binding with various receptors, thus intensifying a series of cellular reactions that are closely related to inflammatory diseases, autoimmune diseases, and cancer (121). Extracellular HMGB1 transmits danger signals to surrounding cells by interacting with its classical receptors. For example, HMGB1 can bind to the receptor for advanced glycation end products (RAGE) (122) and induce inflammation together with Toll-like receptor 2/4/9 (TLR-2/4/9) (25, 123, 124). HMGB1 is released from damaged host cells and activates PRRs (such as RAGE), which upregulates the expression of NLRP3 and IL-1β precursors, activating the NLRP3 inflammasome and binding with IL-1β to exacerbate immune cell-induced inflammation and cellular damage, which then accelerates cancer progression (125, 126).

In recent years, HMGB1 has been studied in ALF, ALI, ACLF, and CLI, although only the relationship between HMGB1 and ACLF is summarized here. HMGB1 is an important proinflammatory molecule in many inflammatory diseases, but its role in ACLF is not completely clear. The existence of HMGB1 is strongly associated with early liver injury, immune activation, and further immune injury during ACLF.

Some clinical experiments showed that increases in serum or tissue HMGB1 levels correlate positively with the occurrence and development of ACLF inflammation. A meta-analysis of 16 studies by Hu et al. revealed that the serum level of HMGB1 in patients with severe HBV and HBV-ACLF is higher than that in patients with mild and moderate hepatitis, and it was speculated that HMGB1 might be an important diagnostic target for ACLF (56). Jhun et al. showed that the expression of HMGB1, RAGE, and IL-17 increased in the liver tissue of severe HBV patients. IL-17 expression induced by the HMGB1/RAGE axis further aggravates the inflammatory reaction of peripheral blood cells in HBV patients, and downregulation of the HMGB1/RAGE axis may effectively reduce the inflammatory reaction (57). According to Cai et al., compared with levels in the healthy control, liver fibrosis, and CHB groups, the serum HMGB1 levels of ACLF patients are significantly increased, suggesting that HMGB1 can provide diagnostic or prognostic information for HBV-related ACLF (58). However, Wu et al. found levels of proinflammatory cytokines to be significantly increased in ACLF patients, whereas serum HMGB1 levels did not change (67).

The mechanism by which HMGB1 participates in the occurrence of ACLF has been partially confirmed. Xu et al. found that HMGB1 mainly exists in bile duct cells. HMGB1 begins to increase gradually at 4 h after stimulation with LPS or TNF-α in cholangiocarcinoma cell TFK-1 culture until the end of stimulation. Due to ischemia and hypoxia, inflammatory stimulation leads to the death of initial hepatocytes and release of HMGB1, demonstrating that HMGB1 plays a key role in the systemic inflammation related to ACLF (59). Gao et al. reported that the protective effect of the recombinant Ad-HGF-HIL-6 adenovirus with hyper-IL-6 and hepatocyte growth factor can significantly reduce serum HMGB1 and other proteins in ACLF rats as well as liver injury and apoptosis activity (61). Xu et al. found that levels of the serum proinflammatory cytokines IL-22, IL-6, and HMGB1 are significantly decreased in ACLF mice treated with a lncRNA-rich transcript-1 (NEAT1)-related adenovirus because NEAT1 blocks TRAF6 ubiquitination in an ACLF rat model to inhibit the inflammatory response (62). Wang et al. reported that ethyl pyruvate significantly improves liver histopathology and reduces levels of serum endotoxin, inflammatory cytokines, and HMGB1 in liver tissue (63); Fang et al. reported that quercetin reduces oxidative stress and apoptosis by inhibiting HMGB1 and its translocation, thus alleviating liver injury in ACLF rats (64); and Yang et al. reported that plasma-soluble T-cell immunoglobulin and mucin-domain-containing molecule-3 (sTim-3) are significantly increased in ACLF and inhibit the release of HMGB1, alleviating inflammatory reactions and liver injury by promoting autophagy and regulating monocyte/macrophage function (65). The latest research results of Hou et al. (2021) revealed that the thermal apoptosis of hepatocytes induced by HMGB1 enhances the inflammatory reaction to aggravate ACLF and that inhibiting HMGB1 *in vivo* obviously improves liver function and coagulation function in ACLF rats, indicating that it is a potential therapeutic target for ACLF treatment (66).

In conclusion, the mechanism of action of HMGB1 in ACLF is not yet fully understood. Blocking the production of HMGB1 with certain drugs or small-molecule preparations can reduce the inflammatory response in the process of ACLF, which may be a new targeted therapeutic strategy for ACLF.

### 3.5 S100 Protein

The S100 protein family is the key mediator for initiating and maintaining inflammation, not only amplifying the initial inflammatory signal and inducing inflammatory reactions but also slowing inflammation, promoting tissue repair, and regulating inflammation, cell proliferation and differentiation, energy metabolism, apoptosis, calcium homeostasis, cytoskeletal functions, and microbial resistance under certain conditions. This family is also becoming a new diagnostic marker for identifying and monitoring various diseases (108). The S100 protein is released by a variety of cells in the inflammatory state to promote the expression of inflammation-related genes and cell damage effects, among which S100A12 was the first S100 protein identified as a RAGE ligand (34). ACLF research has mainly focused on serological experiments, and the mechanism involved remains to be further elucidated.

Early studies revealed that serum S100b levels in fulminant hepatitis patients and ACLF patients are higher than those in cirrhosis patients and normal controls and unrelated to survival time (95). Another study showed that compared with 13 cirrhosis patients without HE and 8 healthy subjects, blood levels of S100b in most of the 35 ALF patients studied were increased but unrelated to the survival rate (93). Later, some scholars found that serum S100b is a useful marker of HE in fulminant hepatitis patients (96). Serum S100A12 may reflect the oxidative stress and inflammation levels of HBV-ACLF patients, and an increase in S100A12 may be an important biological index of poor prognosis (58). Others have found that M2 macrophages can alleviate liver injury and play a protective role in ACLF mice by inhibiting the S100A9 protein-related necrotic inflammation axis, which provides new insight for the treatment of ACLF patients (98). In addition, in children with pediatric acute liver failure (PALF), the serum S100 β level may correlate positively with the severity of the disease (94). Researchers have also found that the serum S100 β level is increased at an early stage in animal ALF models and may be used as a marker of HE (97).

On the basis of clinical experiments, S100 proteins can be used as new potential inflammatory markers of ACLF to assist in the diagnosis or assess the prognosis of ACLF.

### 3.6 Mitochondrial DNA

In recent years, mtDNA, as a DAMP molecule, has been increasingly studied in the context of cell damage. As a proinflammatory mediator, its mechanism in many inflammatory diseases has also been discussed. For example, Tumburu et al. found that mtDNA is a proinflammatory DAMP molecule in sickle cell disease (127) and Todkar et al. reported that mtDNA can enhance the proinflammatory effect in vesicles (116). Zhang et al. reported that mtDNA is greatly increased in systemic inflammatory response syndrome (SIRS), promoting MAPK activity, increasing Ca^2+^ flux and phosphorylation, inducing neutrophil-mediated organ damage, and leading to death in patients by binding with TLR9 and activating human polymorphonuclear neutrophils (PMNs) (117). mtDNA is of concern in genetic diseases and systemic diseases, and its level is related to the severity of inflammation. However, there are few studies on the role of mtDNA in ALF or ACLF.

When hepatocyte damage leads to mitochondrial dysfunction, mtDNA is damaged, consumed, and released into the blood circulation from cells and triggers an inflammatory reaction through TLR9, the inflammatory corpuscle, and the stimulator of interferon genes (STING) pathways, which aggravates hepatocyte damage and even multiple-organ dysfunction (128). McGill et al. reported that compared with survivors, the group of APAP-induced ALF patients who died had higher serum mtDNA levels and that mtDNA could be used as a good biomarker to predict prognosis (99). The mtDNA of DAMPs can be released into the tissue environment by necrotic hepatocytes during liver injury, and free mtDNA can promote the development of inflammation and lead to ACLF by interacting with cyclic GMP-AMP synthase (cGAS) to induce IFN (100). He et al. found that APAP can significantly increase serum mtDNA levels in ALF mice and that mtDNA in necrotic hepatocytes triggers TLR9 on neutrophils, induces expression and infiltration of proinflammatory mediators, and aggravates liver injury (101).

As an inflammatory mediator in the process of hepatocyte injury, mtDNA has received significant attention, but further research is needed to find a stable inflammatory marker for the early diagnosis of ALF or ACLF.

## 4 Summary and perspectives

Currently, liver transplantation is the most effective treatment for ACLF, but its use is limited by the number of donors. Therefore, finding a treatment that limits excessive systemic inflammation without inducing immunosuppression is important for ACLF treatment. The danger signal theory states that the death of tissue cells causes immune cell activation and release of a large number of cytokines, increases immune damage to organs, and eventually leads to systemic inflammation. Early inflammatory mediators such as TNF-α, IL-1β, IL-17, and IFN-γ are important cytokines leading to ACLF, and DAMPs such as HMGB1 and IL-33 have been shown to regulate early inflammatory mediators through the NF-kB signaling pathway. IL-33 enhances the ability of Tc cells to secrete IFN-γ, NK cells to secrete IL-12, and DCs to secrete TNF-α and IL-1β by binding to ST2 on the surface of immune cells. TNF-α and HMGB1 synergistically promote D-galactosamine/LPS-induced acute lethal liver injury, and blocking TNF-α and HMGB1 synergistically improves liver injury (129) while promoting histone acetylation, inhibiting release of a variety of proinflammatory cytokines (TNF-α, IFN-γ, IL-10, IL-18) (53). DAMP-related molecules have been confirmed to be expressed abnormally in ACLF patients, and IL-33, sST2 (28, 30, 32, 106), and HMGB1 (58) have also been found to be single biomarkers that predict the prognosis of ACLF. There is currently no ACLF therapy targeting DAMPs, but neutralization of IL-33 (130) using sST2, HMGB1, and IL-33-blocking antibodies (131–133) and TSA-acetylated histones (53) can effectively reduce the inflammatory cytokine storm caused by DAMPs, which shows the potential of ACLF precision immune diagnosis and treatment targeting DAMPs.

**Figure 2:**
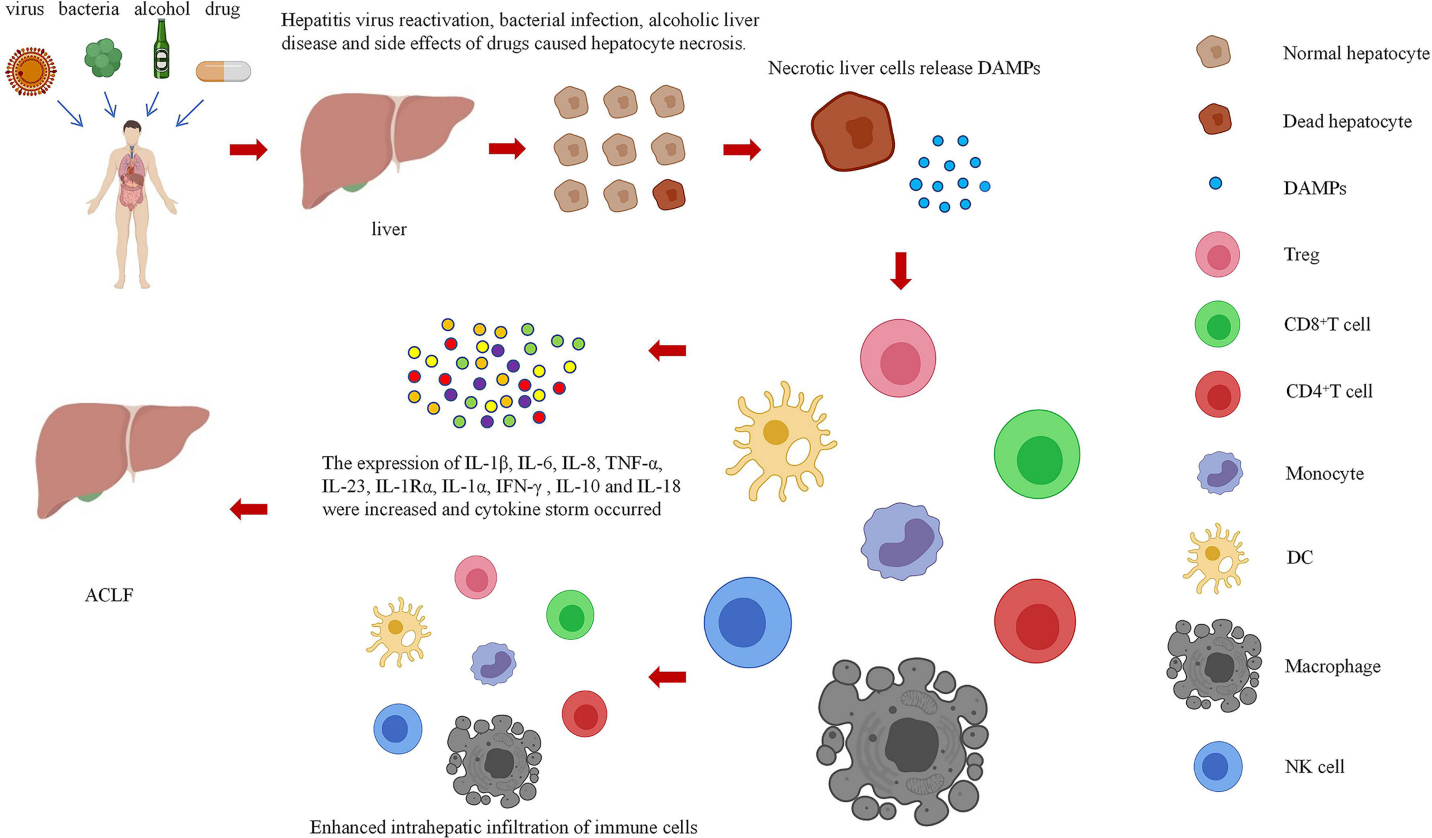
Relationships among cell necrosis, DAMPs, and ACLF.

In this paper, the relationship between DAMPs and ACLF in the danger hypothesis is systematically analyzed, the possible mechanism of DAMPs participating in the immune mechanism of ACLF is explored, and the role of DAMPs in the occurrence of ACLF is studied systematically and deeply by summarizing various methods related to ACLF research. DAMPs mainly result in the occurrence of ACLF by increasing the infiltration of immune cells into the liver and causing a cytokine storm (**Figure 2**). However, the mechanism of these pathways remains unclear, and research is still in the stages of *in vivo* and *in vitro* experiments. Regarding some of the research results, there are still disputes, and the safety and effectiveness need to be further studied. Therefore, it is necessary to further explore the mechanism of the involvement of DAMPs in ACLF.

ACLF is a disease model of systemic inflammation, and studies on the interaction between DAMPs and ACLF will add value to the study of innate immunology and adaptive immune responses that can be extended to other inflammatory diseases beyond ACLF. Furthermore, ACLF serves as a suitable disease model to study the mechanisms of systemic inflammation and tissue damage, as the relationship of each immune cell to DAMPs during ACLF development is poorly understood. With the development of new research tools, such as single-cell RNA-seq and other cutting-edge technologies, the immunological characteristics of ACLF development will be further determined, and how DAMPs affect immune cell polarization and their subsequent effects on ACLF will be revealed. In conclusion, we conducted a literature review of DAMPs and their interactions in ACLF to increase, updating our understanding of this research area and providing new ideas for finding an appropriate immune intervention for ACLF.

## Author Contributions

All authors listed have made a substantial, direct, and intellectual contribution to the work, and approved it for publication.

## Funding

This work was funded by the National Natural Science Foundation (grant number 81900577) and the Science and Technology Plan of Suzhou, China (grant numbers SYS2020192 and GSWS2019067).

## Conflict of Interest

The authors declare that the research was conducted in the absence of any commercial or financial relationships that could be construed as a potential conflict of interest.

## Publisher’s Note

All claims expressed in this article are solely those of the authors and do not necessarily represent those of their affiliated organizations, or those of the publisher, the editors and the reviewers. Any product that may be evaluated in this article, or claim that may be made by its manufacturer, is not guaranteed or endorsed by the publisher.

## Glossary

**Table:**

ACLF	acute-on-chronic liver failure
CTL	cytotoxic T lymphocyte
DC	dendritic cell
DAMPs	damage-associated molecular patterns
ALF	acute liver failure
SALF	subacute liver failure
CLF	chronic liver failure
HE	hepatic encephalopathy
EASL-CLIF	European Association for the Study of the Liver-Chronic Liver Failure
NACSELD	North American Consortium for the Study of End-stage Liver Disease
APASL	Asian Pacific Association for the Study of the Liver
PAMPs	pathogen-associated molecular patterns
PD-1	programmed cell death protein 1
Tregs	regulatory T cells
HLA-DR	human leukocyte antigen-DR isotype;
PRR	pattern recognition receptors
mtDNA	mitochondrial DNA
HSPs	heat shock proteins
HMGB1	high mobility group box chromosomal protein 1
F-actin	fibrous actin
ILC2	type II intrinsic lymphocytes
MyD88	myeloid differentiation factor 88
TRAIL	tumor necrosis factor-related apoptosis inducing ligand
TNF-α	tumor necrosis factor α
FasL	Fas ligand
GalN	D-galactosamine
LPS	lipopolysaccharide
NF	nuclear factor
MAPKs	mitogen-activated protein kinases
ERK	extracellular signal-regulated kinase
Th2	T helper type 2
sST2	soluble ST2
IL-1R	IL-1 receptor
GRP	glucose-regulated protein
GGA	geranylgeranylacetone
APAP	acetaminophen
HSF1	heat shock transcription factor 1
ROS	reactive oxygen species
SOD	superoxide dismutase
BDL	bile duct junction
HAV	hepatitis A virus
UPR	unfolded protein response
PPARα	peroxisome proliferator-activated receptor α
CGA	chlorogenic acid
SMCs	smooth muscle cells
EMT	mesenchymal transition
ALT	alternative lengthening of telomeres
PDHC	pyruvate dehydrogenase complex
LDH	lactate dehydrogenase
CHB	chronic hepatitis B
QCLGF	Qing Chang Li Gan formula
TSA	trichostatin A
HDAC	histone deacetylase inhibitors
RAGE	receptor for advanced glycation end products
TLR	toll-like receptor
NEAT1	lncRNA rich transcript-1
PALF	pediatric acute liver failure
SIRS	systemic inflammatory response syndrome
PMN	polymorphonuclear neutrophils
STING	stimulator of interferon genes
cGAS	cyclic GMP-AMP synthase.
sTim-3	soluble T-cell immunoglobulin and mucin-domain containing molecule-3
